# Nanomaterial-based optical colorimetric sensors for rapid monitoring of inorganic arsenic species: a review

**DOI:** 10.1186/s11671-024-03981-2

**Published:** 2024-02-29

**Authors:** Kalayou Hiluf Gebremedhin, Mebrahtu Hagos Kahsay, Nigus Kebede Wegahita, Tesfamariam Teklu, Berihu Abadi Berhe, Asfaw Gebretsadik Gebru, Amanuel Hadera Tesfay, Abraha Geberekidan Asgedom

**Affiliations:** 1 Department of Chemistry, College of Natural and Computational Science, Mekelle University, Mekelle, Tigray Ethiopia; 2 Department of Environmental Science, School of Environmental Science and Engineering, Tianjin University, Tianjin, China; 3 School of Earth Science, College of Natural and Computational Science, Mekelle University, Mekelle, Tigray Ethiopia

**Keywords:** Colorimetric sensor, Nanomaterial, Toxic metalloid, Arsenic compound, On-site monitoring

## Abstract

Health concerns about the toxicity of arsenic compounds have therefore encouraged the development of new analytical tools for quick monitoring of arsenic in real samples with improved sensitivity, selectivity, and reliability. An overview of advanced optical colorimetric sensor techniques for real-time monitoring of inorganic arsenic species in the environment is given in this review paper. Herein, several advanced optical colorimetric sensor techniques for arsenite (As^+3^) and arsenate (As^+5^) based on doping chromogenic dyes/reagents, biomolecule-modified nanomaterials, and arsenic-binding ligand tethered nanomaterials are introduced and discussed. This review also highlights the benefits and limitations of the colorimetric sensor for arsenic species. Finally, prospects and future developments of an optical colorimetric sensor for arsenic species are also proposed. For future study in this sector, particularly for field application, authors recommend this review paper will be helpful for readers to understand the design principles and their corresponding sensing mechanisms of various arsenic optical colorimetric sensors.

## Introduction

Every year, anthropogenic activities and natural processes emit more than 10 million metric tons of harmful contaminants into the environment [1, 2]. As a result, these toxic contaminants pose a significant risk to human, animal, and environmental safety, which has emerged as a big challenge for the entire world.

Arsenic (As) is a toxic methalloid element widely distributed and is the 20th most abundant element in the Earth's crust, with an average concentration of 2 mg Kg^−1^ [3, 4] Since ancient times, arsenic had been known as one of the most common chemical contaminants in the environment. Millions of individuals are exposed directly, indirectly, or both to this extremely lethal Group-A carcinogen through drinking water and/or other sources [5]. Thus, the main burden on the supply of drinking water is the poisoning of groundwater with toxic compounds, including arsenic species [6]. Arsenic is frequently found in aquatic environments in its many oxidation forms, primarily arsenite (As^III^) and arsenate (As^V^), under a wide range of conditions [7]. Due to its aqueous solubility and unique interaction with sulfhydryl-containing enzymes, arsenite is almost 60 times more hazardous than arsenate [8]. Over the past years, It has been reported that long-term exposure to arsenic contamination in humans is associated with many health issues, including skin lesions [9], cardiovascular disease [10, 11], and a high risk of malignancies [12–14]. Moreover, the accumulation of arsenic species in the soils not only results in contamination of the food chain but also inhibits crop plant growth, which causes losses in crop production [15]. Considering these concerns over arsenic exposure and its widespread occurrence in aquatic environments, the World Health Organization (WHO) and the Environmental Protection Agency (EPA) have set the maximum level of total arsenic concentration in drinking and irrigation water as low as 10 and 100 ppb, respectively [16–18]. Therefore, it is crucial to have an analytical method that is precise, quick, and sensitive for on-site and real-time monitoring of arsenic in drinking water as low as 10 ppb.

Over the past years, several analytical technologies have developed for the detection of arsenic in biological and environmental samples, including blood and urine [19–22]. However, such technologies required either time-consuming sample preparation, expensive equipment, and/or a skilled operator, making them unsuitable for field applications involving real-time monitoring of arsenic species. With the rapid development of nanotechnology, nanomaterial-based signal amplification has great potential for improving the advancement of analytical technologies [23, 24]. The sensitivity and selectivity of the analytical procedures were also further enhanced by the incorporation of nanomaterials and specific-recognition ligands. In fact, significant attention has been drawn to the fabrication of nanomaterial-based optical sensors for the detection of arsenic species by utilizing different recognized ligands such as enzymes, aptamers, and polymers, which are employed directly to bind and react with the target analyte of interest [25, 26]. Specifically, the nano-based colorimetric sensors which require relatively simple designed instruments, on-site application, naked-eye detection, and excellently sensitive recognition made them an effective alternative analytical tool for the detection of arsenic [27–30]. The specific interaction of nanomaterial with arsenic-recognition ligands produces colorimeter signal shifts that enable the accurate detection of arsenic species for visual quantification.

**Fig. 1:**
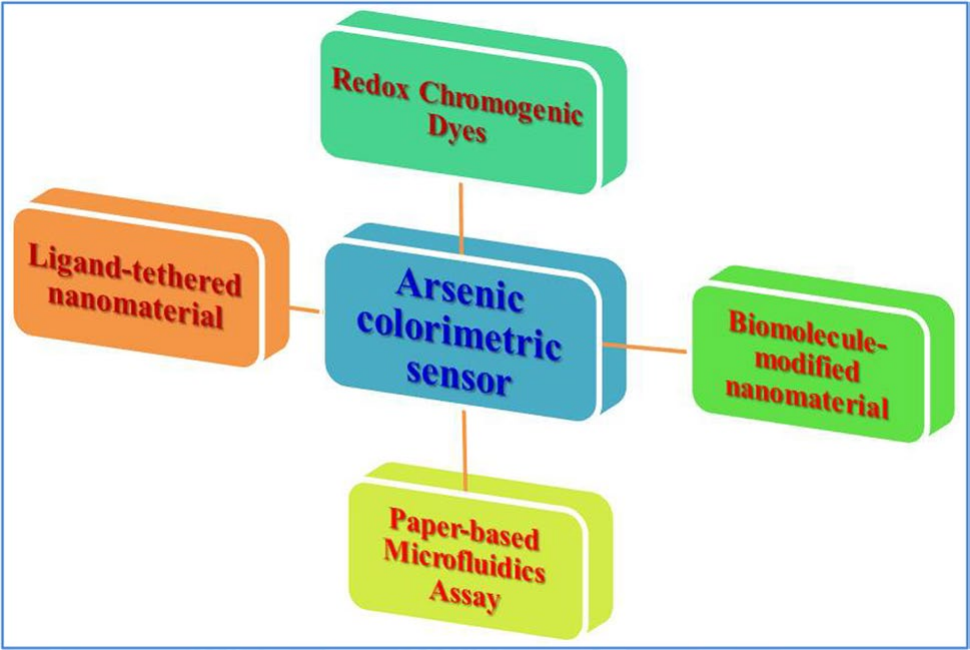
Basic methods of optical colorimetric approaches for arsenic detection

For a better understanding of this novel research field, this review focuses on the recent advancement of an advanced arsenic optical colorimetric platforms based on the inherent redox-chemistry of arsenic, biomolecule-modified nanomaterials, arsenic-binding ligand-modified nanomaterials, and a paper-based microfluidics assay (Fig. 1), which provides comprehensive coverage of the status of arsenic detection. Hopefully, this review could inspire further research in developing novel optical colorimetric sensors for the on-site detection of arsenic in the future and also give some insights on the perspectives and challenges of the nanomaterial modified optical colorimetric sensors design and application for the analysis of arsenic.

## Chemistry and toxicity of arsenic

Due to its enormous health risk, arsenic, a prevalent metalloid pollutant in the aquatic environment, has drawn a lot of attention [31–34]. On this account, several million people are affected by arsenic-contaminated food and drinking water in many countries, such as West Bengal, Bangladesh, the United States, and others [35–37]. In practice, over 200 arsenic-bearing minerals, including elemental, arsenite, arsenate, sulfide, oxide, and arsenide, are identified as the most significant sources of arsenic [38]. In addition to these minerals, arsenic has also been released into the environment from a variety of sources, such as the burning of fossil fuels, volcanic eruptions, arsenic-based insecticides, and industrial activities [39, 40]. These sources could have a significant impact on the pollution of aquatic environments.

Arsenic is an element with the electron configuration [Ar] 3d^10^ 4s^2^ 4p^3^ that is found in four common oxidation states: -3 (e.g., AsH_3_), 0 (e.g., elemental As), +3 (e.g., As_2_O_3_) and +5 (e.g., As_2_O_5_) [41]. These variable redox states indicated that it can react with oxygen, sulfur, nitrogen, and/or carbon to form covalent compounds and generate large numbers of arsenic species [42]. The oxidation potential of elemental arsenic is larger than that of nitrogen and phosphorus, which increases the cationic nature of arsenic and allows it to readily show trivalent and pentavalent oxidation states. For instance, the pentavalent arsenate (As(V) as AsO_4_^−3^) species is the most stable form in oxygenation environments, whereas the trivalent arsenite (As(III) as AsO_3_^−3^) is the dominant form under reducing conditions [43, 44]. The most common arsenite compounds are sodium arsenite, arsenic trioxide, and arsenic trichloride, whereas the most stable pentavalent forms of inorganic arsenic compounds include metal arsenate (e.g., sodium, lead, and calcium arsenate), arsenic acid, and arsenic pentaoxide [45]. Additionally, arsenic is also found in organic form in the environment, and the most common are arsanilic acid, monomethylarsenic acid (MMA), dimethylarsenic acid (DMA), arsenocholine, sodium dimethylarsinate, dithioarsenate, and arsenobetaine [12, 46].

**Fig. 2:**
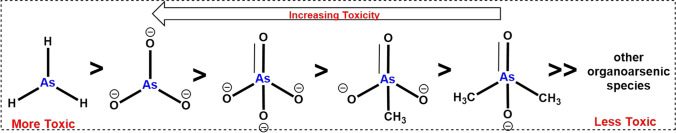
Toxicity pattern of arsenic compounds

The biochemical, environmental, and toxicological characteristics of arsenic compounds largely depend on the oxidation state, solubility, redox potential, and the group or molecule attached to the arsenic [47]. The acute toxicity of arsenic in humans can range from non-hazardous (e.g., arsenosugars) to excessively hazardous (e.g., arsine gas). For instance, arsenite is a very toxic substance that is approximately 60 times more dangerous than arsenate due to its reaction with sulfhydryl-containing enzymes [48, 49]. Organoarsenic compounds are usually considered less hazardous than inorganic arsenics, which are also related to the degree of accumulation in tissues and their rates of absorption and elimination. In sum, the arsenic toxicity pattern is shown in Fig. 2.

The chemistry of arsenic has been involved in many enzyme inhibition processes [50, 51]. As(III), for instance, binding to an enzyme that contains a sulfhydryl group results in inhibiting the enzymatic process as well as other biocellular functions [52, 53]. Because of the structural similarities between As(V) and phosphate, As(V) produces toxicity by mimicking a phosphate group in adenosine triphosphate (ATP) and resulting in interference with the ATP production in mitochondria and destruction of essential metabolic processes. The chronic toxicity of arsenic is associated with a variety of adverse health effects, such as carcinogenesis [54], respiratory problem [55], cardiovascular disease [56], kidney damage [57], and many others [58]. Therefore, it is crucial to detect arsenic early and remove it from drinking water.

## Advanced optical colorimetric strategy for arsenic sensing

**Fig. 3:**
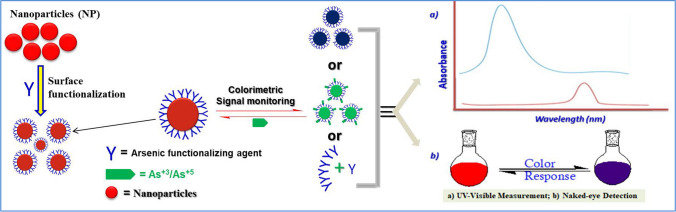
General illustration of basic principle of arsenic-based optical colorimetric nanosensor

The development of optical nanosensors using various functional nanomaterials has shown great potential for the detection of various analytes, including heavy metals [59], pesticides [60], and other chemical toxins [61]. An optical sensor typically has translator components that serve as optical signal indicators and recognition elements that can interact precisely with the target analyte [62, 63]. Due to their high sensitivity, low cost, simple construction, and quick and easy detection with the naked eye, colorimetric approaches are currently attracting a lot of attention among these high-end optical nanosensors [64, 65]. As real-time and on-site monitoring become increasingly crucial, several advanced optical colorimetric sensors have been reported for the detection of arsenic in various sample matrices. In those assays, the molecular and chemical interactions between arsenic compounds and the surrounding materials are the key principle for optical colorimetric sensing mechanisms (Fig. 3). The specific interaction of arsenic with the redox dyes and/or surface-modified nanomaterials results in a color shift in the assay solution that can be seen with the naked-eye without any other sophisticated instruments or with the help of simple equipment such as UV–Vis spectrophotometry [66–68]. The three main categories of the present, well-proven colorimetric-based arsenic sensing strategies are doping redox chromogenic dyes, biomolecule-modified nanomaterials, and arsenic-specific ligand-tethered nanomaterials. In this article, we present a critical review of the application of advanced Optical colorimetric sensing methods for the detection of arsenic ions (As^+3^ and As^+5^) in the environment.

### Sensors based on redox dye chemistry

**Fig. 4:**
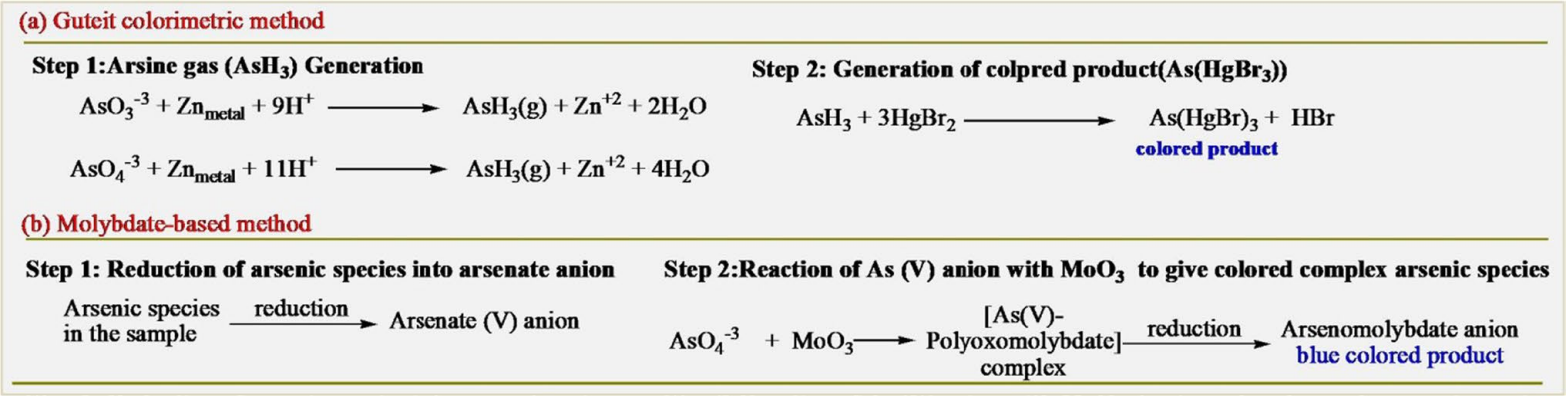
Classical demonstration of optical colorimetric method for arsenic detection. **a** General scheme of the Guteit reaction, **b** General scheme of molybdate-based reaction

Inorganic arsenic species (As^+3^ and As^+5^) have certain inherent redox characteristics (*E*^*0*^_*As(V)/As(III*)_ = 0.56 V) and they can undergo spontaneous redox reactions with a few redox chromogenic reagents [69]. This inherent redox property is used today as the most common colorimetric sensing principle of arsenic for field applications [70–72]. As illustrated in Fig. 4a, there are several commercial colorimetric test kits available for the detection of arsenic based on the Gutzeit reaction [73]. The Gutzeit method has been widely used as a colorimetric field kit technology to test for arsenic in groundwater since 100 years ago [74]. This technique relies on the reduction of arsenic compounds in the sample under acidic conditions with the addition of zinc powder to produce arsine gas (AsH_3_). In order to produce a brightly colored complex that can be measured for quantification, the produced arsine gas was trapped on paper impregnated with different substances, such as mercuric bromide (HgBr_2_) [75], silver diethylthiocarbamate [76], or polyvinyl alcohol/KI [77]. These assays, however, produce extremely toxic by-products that pose a hazard to the analyst and the environment. Additionally, sulfur-containing compounds, like hydrogen sulfide, are found to have potential interference with such colorimetric assay and may require an additional separation or purification step in the procedure.

The development of innovative arsenic colorimetric methods that match or exceed the sensitivity of the Gutzeit method while enhancing selectivity, accuracy, safety, and reproducibility has been the focus of several scientific groups despite these potential drawbacks [78–81]. One possible approach is to use polyoxomolybdate chemistry that reacts directly with arsenic to form a highly colored complex (Fig. 4b) [82]. In this method, first the arsenite (As^+3^) present in the sample effectively oxidizes into arsenate (As^+5^) ions to form an arsenate-polyoxomolybdate complex, which is subsequently reduced to an arsenomolybdate complex, producing a bright blue color solution that can be easily measured using spectrophotometry. For example, Lenoble et al. reported an arsenic-molybdenum complex protocol for measuring low arsenic concentrations in drinking water up to 20 ppb detection limit [83]. This method is relatively fast and safe, and there is no major interference from silicate and sulfate up to 10–30 ppm concentrations, while iron and phosphate still cause a significant amount of interference. To minimize the above concerns, several research groups have developed a pre-treatment step using ion chromatography and an ion-exchange column to concentrate and isolate arsenic from the rest of the sample prior to the arsenate-molybdate complex formation [84]. Both methods employ the chromatography method either before and/or after all of the sample's arsenic compounds have been converted to arsenate. After eluted from the column, the arsenate ion interacts with molybdenum oxide to produce the arsenate-molybdate complex, which is subsequently analyzed and quantified. Thus, the separation technologies could improve the sensitivity and achieve a lower than 8 ppb detection limit for the total arsenic.

In recent years, Das and Sarkar developed a ploymer hydrogel-based, cost-effective colorimetric strip sensor for arsenate detection based on the reduction reaction between the arsenate ion and ammonium molybdate [85]. The strip was fabricated by encapsulating ammonium molybdate in a polymer hydrogel made of polyvinyl alcohol, glutaraldehyde, and acrylamide. This assay was constructed on the formation of a blue-colored arsenomolybdate complex under reductive conditions, which can be seen by the naked eye down to the 10 ppb detection limit [85]. This technique is easy to use and precise, and it thus may be used to analyze arsenic in real environmental samples. Furthermore, many research groups have explored the quantitative analysis of arsenic ions by forming the arsenomolbdate complex using a simple spectrophotometer [86].

**Fig. 5:**
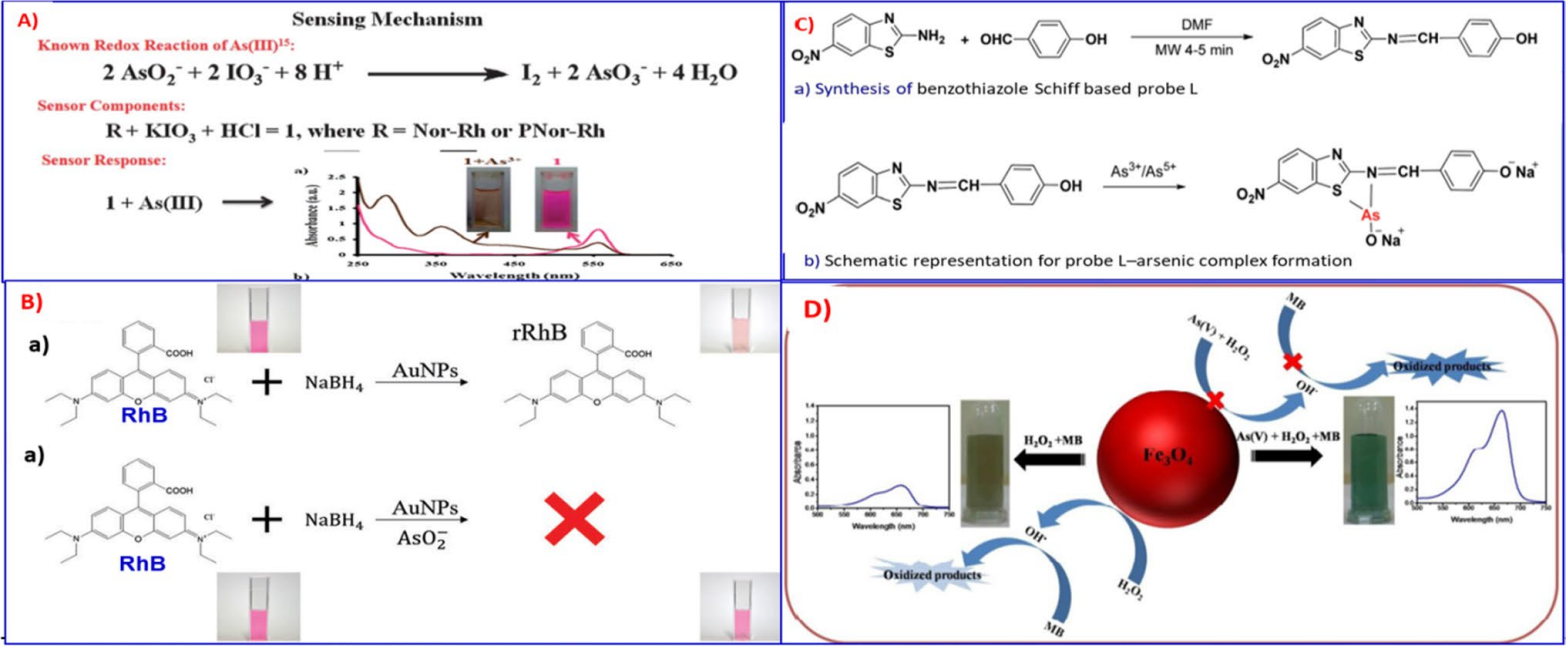
**A** Schematic representation of Norbornene polymer-based arsenic detection, adapted from Ref. [87]. **B** Schematic representation of a B (RhB)-based sensor for arsenic sensing before (a) and after (b) the addition of AsO_2_^−^, adapted from Ref. [88]. **C** Schematic representation for benzothiazole Schiff-based probe for arsenic As(III)/As(V) detection, adapted from Ref. [89]. **D** A schematic diagram illustrates the Fenton-like mechanism involved in the colorimetric detection of As (V) by Fe_3_O_4_ nanoparticles, adapted from Ref. [90]

However, in order to minimize potential interference from iron and phosphate, the majority of the complex arsenomolbdate techniques required complicated separation procedures. To address the above limitations, some research groups aimed to concentrate on dye-based chemistry to demonstrate superior performance for the determination of arsenic [91, 92]. In this case, arsenic compounds directly bind to or interact with dye molecules in order to induce structural and colorimetric variations that can be easily measured and quantified. For example, Bhattacharya et al. fabricated a norborene derived rhodamine monomer (Nor-Rh) sensor based on redox chemistry for selective and sensitive detection of arsenite in the presence of iodate in an acidic solution [87]. Arsenite and iodate can spontaneously undergo a redox process in which iodate is reduced to iodine and arsenite is oxidized to produce arsenate (Fig. 5A). This redox reaction is very specific to the arsenite ion. The norbornene-I2 adduct can be formed by interacting the product iodine with the norbornene-Rh dye. The formation of halogen bonding between the carbonyl oxygen of rhodamine and the adduct norbornene-I2 resulted in a unique optical property change. This approach is able to sense arsenite at the ppb level selectively when it is passed through a column backed by Nor-Th polymers. By observing these colorimetric and fluorescence features, arsenite (As^+3^) can thus be detected in the sample.

Similarly, Xu et al. (2018) reported ultrasensitive colorimetric nanoprobes for on-site testing of arsenic using an unmodified gold nanoparticle (Au NP) and Rhodamine-B (RhB) as a color signal reporter (Fig. 5B) [88]. This assay is based on the inhibition of the Au NP catalytic-redox reaction between RhB and sodium borohydride by AsO_2_- ion, which results in a color and absorption peak change. This assay exhibits great selectivity against other interfering ions and high sensitivity, with a limit of detection of 0.64 ppb, which is below the WHO-recommended level for arsenic in drinking water.

Another promising benzothiazol-based Schiff base probe was also reported for on-site detection of arsenite and arsenate at low concentrations in aqueous media [89, 93]. A microwave-based condensation reaction was used to synthesize the benzothiazole-based Schiff base in one-step (Fig. 5C). The proposed colorimetric probe results in a rapid visible color change from pale-yellow to dark orange due to the bis-coordination of As^+3^ or As^+5^ to the N, S chelate of the benzothiazole-based Schiff base probe. This colorimetric assay provides several advantages over the classical Gutzeit approach, including simplicity, low cost, high sensitivity, excellent selectivity, and specificity. Toluidine Blue [94], Malachite Green [95] and other dye-based colorimetric techniques were also investigated for the detection of arsenic. More significantly, because of the quick reaction time, Fenton-like catalytic reactions have been widely used in colorimetric sensors, and thus they can be used for arsenic sensing platforms. Using methylene blue (MB) as a color signal indicator, for instance, Christus and his group developed a novel sensitive colorimetric sensor for the analysis of arsenate in a water sample based on the Fenton-like reaction of Fe_3_O_4_ nanoparticles [90]. Due to the production of a hydroxyl radical via a Fenton-like reaction, the produced Fe_3_O_4_ nanoparticle catalyzed the oxidation of MB dye into a colorless solution (Fig. 5D). Moreover, the addition of arsenate can be bound on the active site of Fe_3_O_4_ NP, where it inhibits the catalytic activity of Fe_3_O_4_ NP and restores the blue color. The method shows a good detection limit value of 0.358 nM in natural water. All of these dye-based colorimetric assays are limited to detecting organoarsenic species such as MMA and DMA.

### Biomolecule-modified nanosensors

#### Sensor-based on enzyme mediated reaction

Many arsenic compounds, like other toxic pollutants, block or inhibit some natural enzyme activities that are important in toxicology and pharmacology, including laccase, acid phosphatase, peroxidase, and others [96–98]. Arsenic poisoning is caused by either the inhibition of sulfur-containing enzymes or the inhibition of phosphorylation activity [99]. The concentrations of arsenic are often measured by quantifying the percentage of enzyme activity inhibition before and after exposure to the arsenic species (inhibitor) [97]. By integrating the enzyme-mediated inhibition principle with nanomaterials as a signal reporter, therefore, it is now possible to construct and develop new optical colorimetric tests for detecting arsenic by assessing enzyme activity inhibition [100]. In principle, the appropriate substrate can be hydrolyzed into products in the presence of enzymes, resulting in a change in the analytical signal indicator. When an additional arsenic compound is added, the catalytic activity of the enzyme is inhibited or blocked, resulting in the recovery of the analytical signal indicator by increasing the concentration of arsenic.

So far, only a few contributions have been made to the colorimetric detection of arsenic species based on enzyme inhibitory effects rather than other optical or electrochemical biosensors. For example, Zhang et al. constructed a plasmonic light-up colorimetric biosensor for arsenate detection in groundwater by aggregating gold nanoparticles (Au NP) based on the inhibitory effect of arsenate on acid phosphatase (AcP) bioactivity [101]. The substrate for AcP in this approach was adenosine 5'-monophosphate (AMP). The AcP catalyzes the hydrolysis of negatively charged AMP to uncharged adenosine, resulting in significant aggregation of Au NPs and a shift in the color of the solution from red to blue. Arsenate can efficiently reduce AcP enzymatic activity by competitive inhibition, which causes a slowdown of the catalytic hydrolysis process. This is confirmed by the color change from blue to red with increasing concentrations of arsenate. Similarly, Wang et al*.* proposed a self-powered biosensor for arsenite and arsenate detection based on reversible inhibition of laccase activity [102]. Laccases are Cu-containing enzymes that have been shown to be capable of both mediated and direct electron transfer [103]. Enzymatic inhibition of laccase has been demonstrated using a colorimetric assay with 2,2'-azino-bis(3-ethylbenzothiazoline-6-sulfonic acid). Laccase was then linked with FAD-dependent glucose dehydrogenase in a series of chemical modifications to create laccase-based bioelectrodes that can use as a self-powered biosensor for both arsenic species detections. This method achieved a detection limit of 13 µmol/L for arsenite and 132 µmol/L for arsenate. Despite the benefits of these enzyme-based sensors, significant issues emerge when they are applied to environmental samples.

Nanosized materials with intrinsic enzyme-like activity, defined as nanozyme, exhibit superiorities in excellent stability, low cost, adjustable catalytic activity, and ease of large-scale production [104–106]. Several nanozymes have been shown in recent years to have enzyme-mimicking catalytic properties similar to natural bio-enzymes, such as peroxidase and oxidase-like activities [107]. This technology is innovative in that it integrates numerous technologies to construct an arsenic colorimetric nanosensor based on enzyme-mimicking activity inhibition [106, 108]. Several nanozyme materials made of noble metals, transition metals, and their derivatives have shown promise in the optical detection of harmful contaminants [109]. However, there are few reports about peroxidase-like nanozyme-based colorimetric probes for the detection of arsenic in the environment [110, 111].

**Fig. 6:**
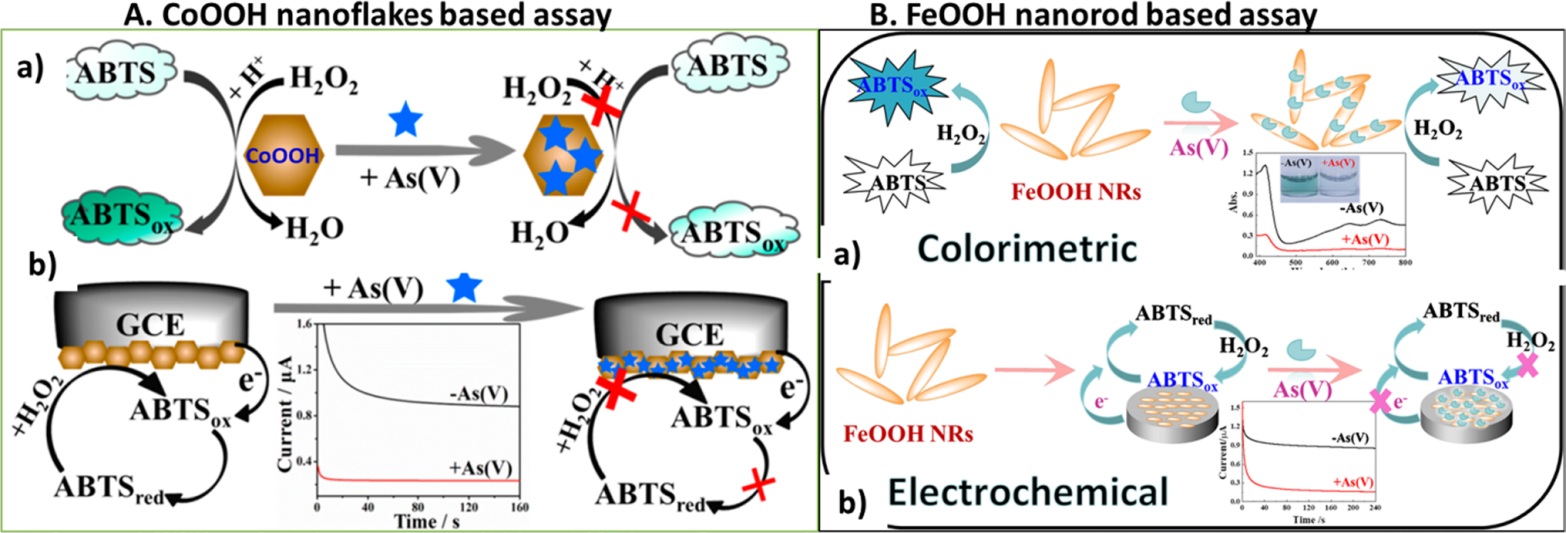
Schematic demonstration of As (V) controlled Peroxidase-like activity. **A** CoOOH nanoflakes based assay for detection of arsenate, adapted from Ref. [110], and **B** FeOOH nanorod-based assays for detection of arsenate, adapted from Ref. [111]

Wen et al*.* reported that cobalt oxyhydroxide (CoOOH) nanoflakes possessed peroxidase-like activity with 2,2′-azino-bis(3-ethylbenzthiazoline-6-sulfonic acid) (ABTS) in the presence of hydrogen peroxide (H_2_O_2_) for arsenate detection [110]. Interestingly, CoOOH nanoflakes can catalyze the conversion of ABTS substrate into green oxidized ABTS product in the presence of H_2_O_2_. In this work, arsenate can specifically bind with CoOOH nanoflakes via AS-O bond interaction and electrostatic attraction (Fig. 6A), which inhibit the peroxidase-mimic activity of CoOOH. Thus, a simple colorimetric probe was developed based on arsenate-specific inhibition of the CoOOH nanozyme toward ABTS catalysis with a detection limit of 3.72 ppb. Furthermore, the CoOOH nanozyme-ABST chromogenic substrate system was employed for very sensitive electrochemical sensing of arsenate in ambient samples, with a detection limit of 56.1 ppb. Similarly, Zhong et al. developed a dual colorimetric and electrochemical test for arsenate detection by utilizing the peroxidase-like activity of iron oxyhydroxide (FeOOH) nanorods with ABTS as a chromogenic agent [111]. The FeOOH nanorods were produced using a hydrothermal technique and have outstanding peroxidase-like activity, allowing them to rapidly oxidize the colorless ABTS substrate into a dark green product (ABTSox) in the presence of H_2_O_2_ (Fig. 6B). Arsenate can be adsorbed on the surface of FeOOH nanorods, establishing a strong As-O link and inhibiting the peroxidase-like activity of the produced nanorods, resulting in a decrease in green product oxidation. This method was also used to successfully identify arsenate in environmental water samples, with detection limits of 0.1 ppb and 12 ppb for colorimetric and electrochemical assays, respectively. However, these approaches have significant drawbacks, such as the possibility of phosphate ion interference. However, these approaches have significant drawbacks, such as the possibility of phosphate ion interference. Furthermore, the presence of H_2_O_2_ as a substrate for peroxidase-like activity can result in the production of oxygen free radicals as by-products, affecting the stability and accuracy of measurements.

**Fig. 7:**
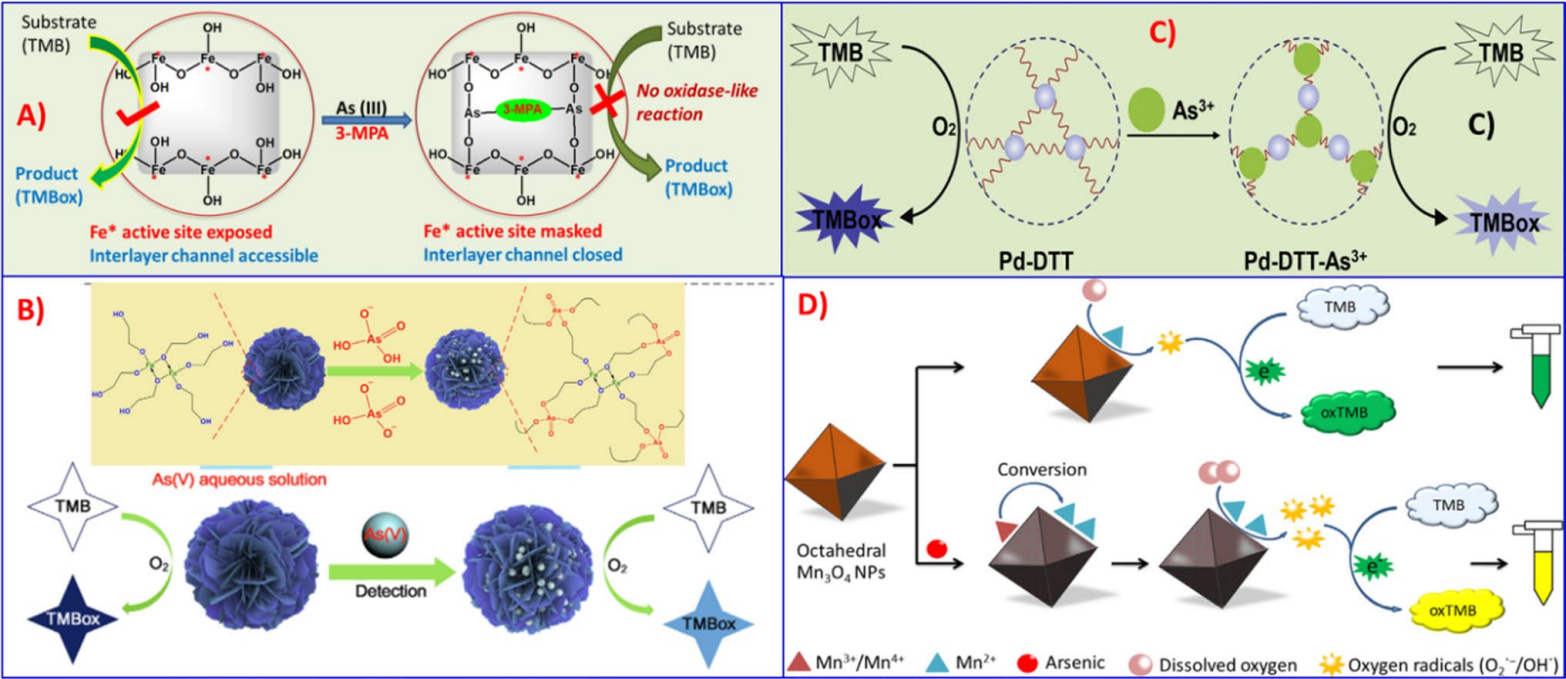
Schematic demonstration of Oxidase-like activity based colorimetric assay for arsenic detection. **A** Illustration of the dual-masking of the active site and interlayer channel of the oxidase-mimicking Fe-Co-LDH by arsenite with the assist of 3-MPA, adapted from Ref. [112]. **B** Three-dimensional flower-like iron alkoxide based nanozymes for colorimetric detection of As(V), adapted from Ref. [117]. **C** Illustration of the reassembly-induced oxidase-mimicking activity inhibition of Pd-DTT for colorimetric analysis of arsenite, adapted from Ref. [114]. **D** Schematic illustration of the colorimetric sensor for arsenic detection based on adsorption enhanced catalytic activity of the octahedral Mn3O4 NPs, adapted from Ref. [115]

Since oxidase-like nanozyme materials do not require H_2_O_2_, they are a more attractive material for sensor design technology than peroxidase-mimicking activities. To overcome this limitation, Fe-Co-hydroxide nanozymes [112], iron alkoxide [113], Pd nanozymes [114], and Mn_2_O_3_ nanoparticles [115] have been exploited recently based on oxidase-mimicking activity inhibition for the development of ultrasensitive colorimetric detection of arsenic. For example, Xu et al. published a colorimetric test for the selective detection of arsenite based on the dual-masking of the active site and interlayer channel of the oxidase-like activity of Fe-Co-layered double hydroxide (Fe-Co-LDH) with the help of 3-mercaptopropinic acid. (3-MPA). The Fe-Co-LDH nanozyme was developed to work as an oxidase-mimetic catalytic activity, promoting the oxidation of colorless 3,3',5,5'-tetramethylbenzidine (TMB) to its blue product (TMBox). More importantly, the sulfhydryl and carboxyl groups in 3-MPA can assist As(III) in being anchored onto the oxidase-like Fe-Co-LDH through the formation of the sulfur-stable Fe-As(III)-3-MPA-As(III)-Fe structure (Fig. 7A), which results in not only chemically masking the Fe* catalytic active site but also blocking the interlayer channel of Fe-Co-LDH for TMB substrate access. Because of the selective dual masking of Fe-Co-LDH nanozyme, the oxidase-like chromogenic reaction of TMB is blocked, and nearly no color change occurs. The method was applied to detect arsenic in water samples with a limit of detection of 35 ppb.

Similarly, Fe-based nanozymes such as Fe_3_O_4_, Fe_2_O_3_, and iron alkoxide have been used to explore many biomedical applications based on their enzyme-mimicking activity [116]. Among them, iron alkoxide nanoparticles were proved to possess oxidase-like catalytic performance for the colorimetric detection of arsenic by utilizing TMB as a chromogenic substrate [113]. Three-dimensional flower-like iron alkoxide-based nanozymes for colorimetric detection of As(V) were illustrated in Fig. 7B with a 1.57 µg/L visual detection limit for As(V). Furthermore, a dithiothreitol-capped palladium nanoparticle (Pd-DTT) was designed based on the reassembly-induced oxidase-like activity for very sensitive colorimetric detection of arsenite [114]. The Pd-DTT nanozyme has strong oxidase-like catalytic activity in the presence of dissolved oxygen, causing the oxidation of colorless TMB to generate the oxidized blue product TMBox. When the arsenite (As + 3) ion is added, it can strongly chelate with the sulfdryl group in DTT, resulting in the reassembly of Pd-DTT nanozymes and, as a result, a considerable reduction of the TMB chromogenic process (Fig. 7C). Based on this strategy, Pd-DTT nanozyme achieved highly sensitive detection of the As^+3^ ion with an ultralow detection limit of 35 ng/L and a wide linear range from 33 ng/L to 333.3 µg/L.

Recently, the affinity of manganese oxide (Mn_3_O_4_) nanoparticles towards arsenic was also investigated based on the adsorption-enhanced oxidase-mimicking catalytic activity of Mn_3_O_4_ NPs [115]. The octahedral Mn_3_O_4_ NPs possess oxidase-like catalytic activity and can catalyze the reduction of oxygen into free radicals. The free radicals can further oxidize the colorless TMB substrate into a blue-green product (Fig. 7D). Upon addition of arsenic solution, interestingly, the surface morphology of Mn_3_O_4_ NPs has been changed by the adsorption of arsenic, which enhances their oxidase-like catalytic activity. Consequently, the solution changes to yellow and displays excellent sensitivity and selectivity against other metal ions. The colorimetric chemosensor can visually detect arsenic with a limit of detection of 1.32 µg/L. Compared to natural enzyme based biosensors, these nanozyme-based colorimetric sensors have advantages that include portability, specificity, sensitivity, and cost-effectiveness. They are also applicable for reliable assessment of total arsenic in complex environmental monitoring.

#### Colorimetric sensor-based on aptamer

Oligonucleotides, defined as aptamers, have emerged as bio-recognition elements via the systematic evolution of ligands by experimental enrichment (SELEX) technology to form a sequence library [118, 119]. Aptamer-based optical biosensors have drawn notable attention for the detection of toxic pollutants, specifically metal ions, because many metal ions exhibit a high affinity for single-stranded DNA and RNA [104, 120]. Regarding the detection of arsenic, Kim and co-workers, who used it for the removal of arsenic from ground water in Vietnam in 2009, had successfully screened an arsenic-binding DNA aptamer, and named Ars-3 aptamer [121]. Based on this, many aptamer-based biosensors have been developed using the Ars-3 aptamer for arsenic detection in environmental monitoring [120, 122]. Like the "lock-and-key" model, aptamers usually undergo molecular and structural shape complementarities, which result in some analytical signal changes, providing an interesting sensing strategy for real-time and on-site detection of arsenic in real samples.

**Fig. 8:**
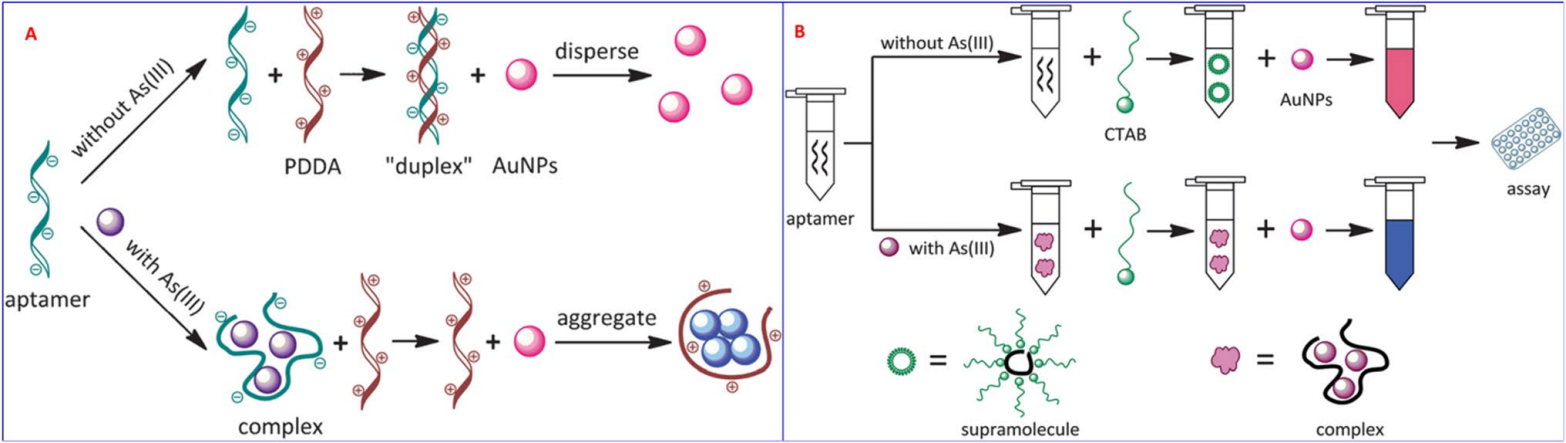
**A** Schematic demonstration of the biosensor for As(III) detection based on the PDDA-induced aggregation of AuNPs, adapted from Ref. [125]. **B** Schematic illustration of the biosensor for As(III) detection based on the CTAB-induced aggregation of AuNPs, adapted from Ref [126]

Among the optical aptasensors, the AuNPs-based colorimetric method is a new and powerful strategy because of its easy and convenient fabrication, distinct size-dependent color formation, and high extinction coefficients [123, 124]. For the first time, Zhou and co-workers reported an aptamer-based colorimetric assay for detecting trace levels of As (III) in aqueous solution [125–128]. A sensitive and selective colorimetric aptasensor was developed by modulating the aggregation of AuNPs with Ars-3 aptamer and cationic polymers (poly(diallyldimethylammonium), PDDA) for the detection of As (III) in aqueous solution [125]. Without As(III), the PDDA can bind with the Ars-3 aptamer to form a "duplex" structure via electrostatic interaction, and thus the subsequent AuNPs cannot aggregate without color change (Fig. 8A). On the addition of As(III), some of the base of the Ars-3 aptamer will react with As(III) to form an aptamer-arsenic complex, leading to aptamer conformation evolution. Thus, the free PDDA can mediate the aggregation of AuNPs with color and absorption spectra changes.

**Fig. 9:**
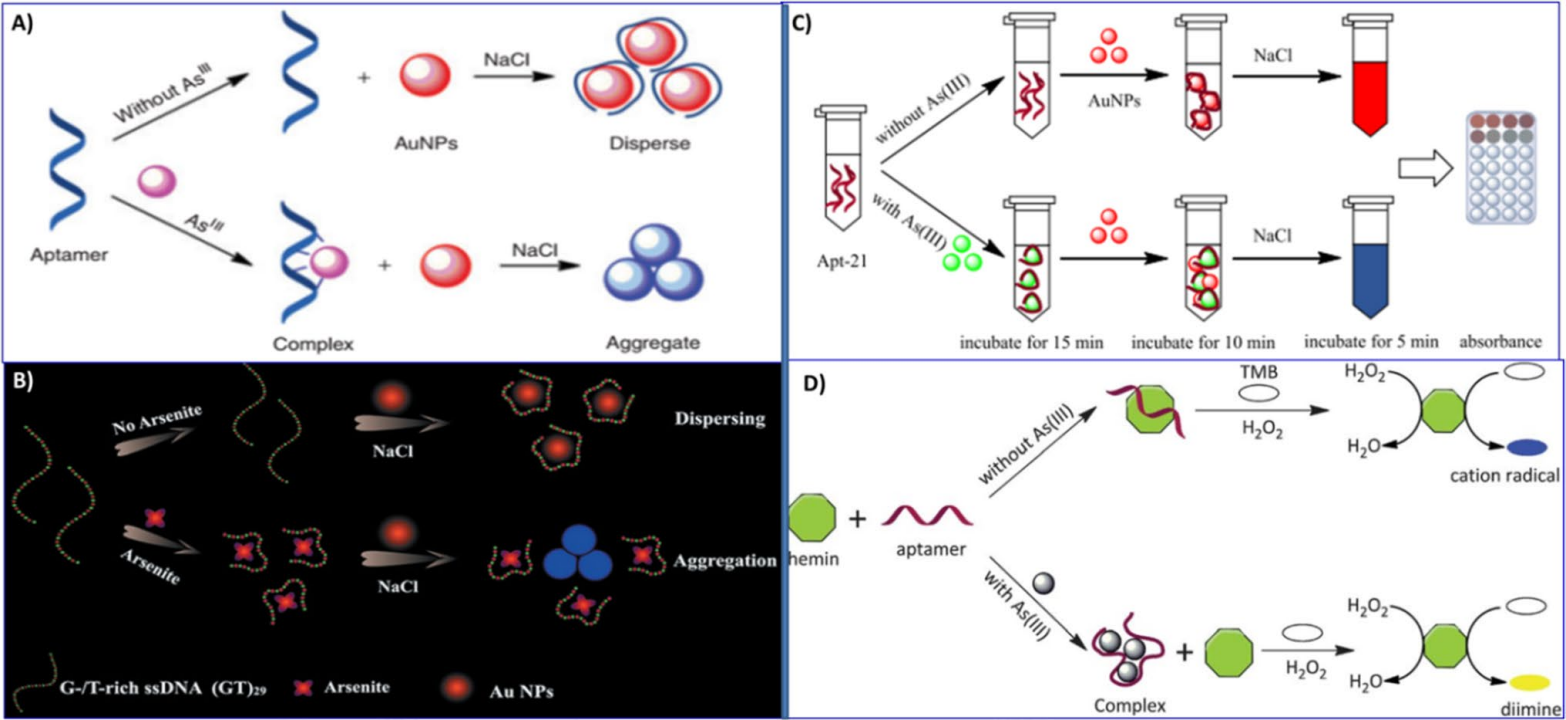
**A** Schematic demonstration of the colorimetric assay for trace arsenic(III) detection in aqueous solution based on arsenic aptamer and gold nanoparticles, adapted from Ref. [127]. **B** G-/T-rich ssDNA Au-NP-based colorimetric strategy for arsenite detection, adapted from Ref. [130]. **C**. Mechanism of the colorimetric determination of As(III) based on short ssDNA and AuNPs, adapted from Ref. [131]. **D** Schematic description of the colorimetric detection of As(III) based on the inhibition of hemin peroxidase activity by arsenic-binding aptamers adapted from Ref [128]

Using the same strategy, they also reported another aptamer-based colorimetric sensor for arsenic detection by replacing PDDA with another surfactant (hexadecetyltrimethyl-ammonium bromide, CTAB) in the same year [126]. Compared to the PDDA agent, CTAB causes the formation of an orderly arranged aptamer molecular bilayer on the surface of AuNPs, which offers very high specific interactions between the aptamer and the As(III) ion with a high degree (Fig. 8B). It also exhibited rapid response and high selectivity over other metal ions, with excellent recovery in a real water sample. The replacement of PDDA by a CTAB binding agent leads to a further improvement in the limit of detection, from 5.6 ppb to 0.6 ppb for color analysis. Similarly, a synergistic molecular assembly of aptamers and CTAB induced AuNPs aggregation based on an aptamer colorimetric biosensor for arsenite detection was also reported by another research group using the same surfactant-induced aggregation of AuNPs [129]. Despite the advantages of this naked eye detection of arsenic, the design of competitive binding of PDDA/CTAB ligands made the sensing system very complex when applied to the biological samples. Furthermore, salt-induced aggregation of a bare AuNPs-based colorimetric aptasensor for arsenic detection was also reported by Zhou's group in 2014 [127]. In this study, an Ars-3 aptamer was employed as a sensing probe and AuNPs as a colorimetric reporter. Without the As(III) ion, the unmodified AuNPs were wrapped by the random-coil Ars-3 aptamer through DNA base-Au interactions that maintain the AuNPs dispersion in the solution even at higher NaCl concentrations, resulting in a red-colored solution (Fig. 9A). In the presence of As(III), the As (III) binds to the Ars-3 aptamers to form an As-aptamer complex. Under this condition, there is no free Ars-3 aptamer that can be adsorbed onto the surface of bare AuNPs. This process fully induces the aggregation of AuNPs in NaCl solution and forms aggregates, accompanied by a change in color from red to blue. By monitoring the color variation, a very rapid classical aptamer-based AuNPs colorimetric method for As(III) was demonstrated with a 1.26 µg/L limit of detection for a dynamic range from 1.26 to 200 µg/L. However, a bare AuNPs-based biosensor was dependent on salt-induced aggregation, which seems to make them more susceptible to potential interference during environmental sample analysis. Additionally, the use of full-length, 100-nt arsenic-binding aptamers in these assays leads to a total increase in the detection costs.

Similarly, label-free arsenic colorimetric assays were also developed based on salt-induced aggregation of AuNPs By using G-/T-rich ssDNA [130] and truncated short ssDNA [131] as sensing element. Liang et al*.* developed a more economic and much simpler label-free colorimetric method for As(III) detection based on the different adsorption on AuNPs between uncoiled G-/T-rich ssDNA and folded DNA [130]. Due to the coordination between Au and nitrogen atoms of bases in DNA, in this assay, the adsorption of uncoiled G-/T-rich ssDNA on AuNPs can prevent the NaCl-induced aggregation of AuNPs and result in maintaining the red color of the colloid AuNPs solution (Fig. 9B). As(III) can particularly form hydrogen bonds with the bases in G-/T-ssDNA. The desorption of DNA from the surface of AuNPs caused the aggregation of AuNPs and a distinct visible color change from red to blue in the presence of salt. This was caused by the conformation evolution of ssDNA from an uncoiled structure to a folded compact structure. The detection of As(III) by using this approach is sensitive, selective, and cost-effective, which compares favourably with previously reported colorimetric methods.

However, this method has also limitations, such as irreversible salt-induced aggregation and slow kinetics between As(III) and G-/T-rich ssDNA. Moreover, Zhang and co-workers developed an unlabeled colorimetric sensing system for As (III) detection based on truncated short ssDNA (Apt-21) from the parent As (III)-aptamer [131]. In this work, Apt-21 functioned as a trace-level assay for As(III) recognition and control of AuNP dispersion by surface attachment, while AuNPs served as a colorimetric signal indicator (Fig. 9C). Another route was reported based on the regulation of hemi-peroxidase catalytic activity by arsenic-binding DNA aptamers. Wu et al*.* reported a novel colorimetric aptasensor for As(III) determination based on the oxidation of TMB by the hemi-peroxidase-H_2_O_2_ system [128]. In the absence of As (III) ions, Ars-3 aptamer binds to the pyrrol ring of hemin via π-π stacking interaction, resulting in temporary inhibition of the catalytic activity of the hemin-H_2_O_2_ system and oxidation of TMB into a blue product of a radical cation. But on As(III) addition, Ars-3 aptamer forms an As(III)-aptamer complex. Thus, hemin regains its catalytic activity and oxidizes TMB to yield the yellow-colored product diimine (Fig. 9D). Through this approach, a detection limit of 6 ppb was achieved with high selectivity against other metal ions. Although these nanosensors displayed high sensitivity to the environment, their disadvantage could not be neglected, as they are highly dependent on the nanoparticle’s aggregation mechanism.

In addition to AuNPs-based colorimetric sensors, other important nanozyme materials exist, such as silver nanoparticles [132], Titanium Dioxide [133] and carbon nanotubes (CNTs) [134], have also been employed for the detection of trace arsenic in drinking water.In particularly, Divsar et al*.* fabricated an AgNPs-based colorimetric aptasensor (Apt-AgNPs) for colorimetric sensing of arsenic in aqueous solutions [132]. The sensing principle is based on the aggregation of Apt-AgNPs by arsenite ions. As(III) could selectively bind with its aptamer to form the As(III)-Apt-AgNPs complex and result in a clear decrease in absorption intensity (λ_max_ = 403 nm), which can be in direct proportion to the arsenite concentration present in the solution. Further, a central composite design method combined with response surface methodology was employed for optimizing the colorimetric efficiency of As(III) analysis. The method demonstrated a linear range of the As(III) concentration from 50 to 750 mg/L with a detection limit of 6 mg/L. The combination of carbon nanotube and Chistosan-Nafion (Chit-Naf) composite has also been studied for rapid monitoring of arsenic in the environment.

**Fig. 10:**
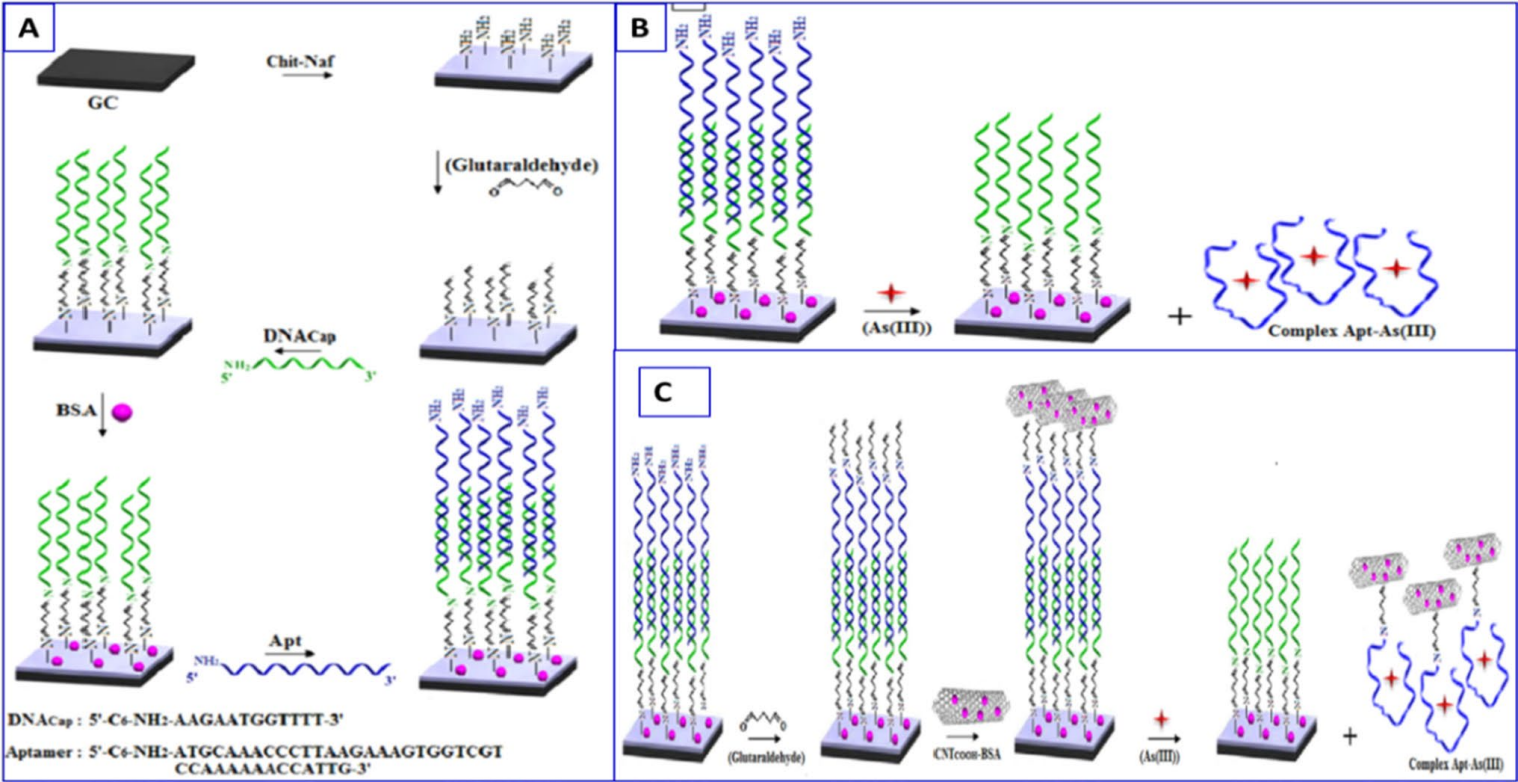
**A** Schematic representation of the aptasensor fabrication steps; **B** Schematic representation of the impedimetric analysis of As(III) by the present aptasensor; and **C** Schematic representation of the various amplification steps of the aptasensor in the presence of CNT_COOH_-BSA hybrid system, reproduced from Ref .[132]

For instance, Salehi et al. modified a glassy carbon electrode (GCE) with Chit-Naf to achieve high electron transfer kinetics and a large immobilization site for the arsenic-binding aptamer, which promise great potential for the fabrication of biosensing assays [134]. The five steps of aptasensor fabrication were demonstrated, as shown in Fig. 10A. In this study, Chit-Naf composite was used as an excellent conductive surface platform, and a novel CNT-based signal amplification process was developed to design a label-free impedimetric aptasensor for As(III) detection with high sensitivity. In the presence of As (III), As (III) induced the dissociation of the aptamer molecule from the sensing interface to form the aptamer-As(III)-complex (Fig. 10B). The high affinity of an As(III)-specific aptamer as the recognition layer for As(III) resulted in a highly selective aptasensor for the detection of As(III) in environmental samples with high accuracy. For As(III) signal amplification, a CNT_*COOH*_-BSA conjugate based on bovine serum albumin (BSA) and CNT was employed as signal amplification (Fig. 10C). The detection limit of this aptasensor for As(III) detection was obtained to be 100 Ω nM^−1^. Although these strategies have high selectivity and sensitivity for As(III), they also suffer from many shortcomings, such as being highly costly, time-consuming, having low stability in harsh environments, and requiring an interference-eliminating process.

#### Sensor-based on other biomolecule-modified nanomaterials

Recently, a protein-based sensing approach has also been employed for developing optical sensors for detecting metal ions. Metal ions can specifically interact with sequenced short peptides that can induce or prevent the aggregation of gold nanomaterials [135]. The complexation of As(III) with a phytochelatine-like peptide is reported to bind arsenic to form a label-free colorimetric assay based on the prevention of thiol-induced aggregation of unmodified AuNPs upon the addition of arsenite solution. This phytochelatine-based As(III) sensor showed simplicity and high sensitivity. However, this assay also responded to As(V) because thiol-rich proteins can reduce As(V) to As(III). Similarly, Chen et al*.* (2017) reported a highly selective colorimetric sensor for detecting As(III) using a highly binding peptide sequence (T-Q-S-Y-K-H-G) as the As(II) specific-recognition moiety [136]. The rational design of the T-Q-S-K-H-G-Cysteine peptide sequence induces the aggregation of unmodified AuNPs, whereas it prevents the peptide-induced aggregation of unmodified AuNPs upon the addition of arsenic solution. This assay shows high selectivity over other metal ions with a LOD of 54 nM for As(III) and was applied for the analysis of As(III) in environmental water samples. This sensing system is much more simple compared with previous reported methods and also features a high selectivity of As(III) over As(V), offering a potential for the speciation of arsenic.

### Sensor-based on specific interactions with ligand-tethered nanomaterials

**Fig. 11:**
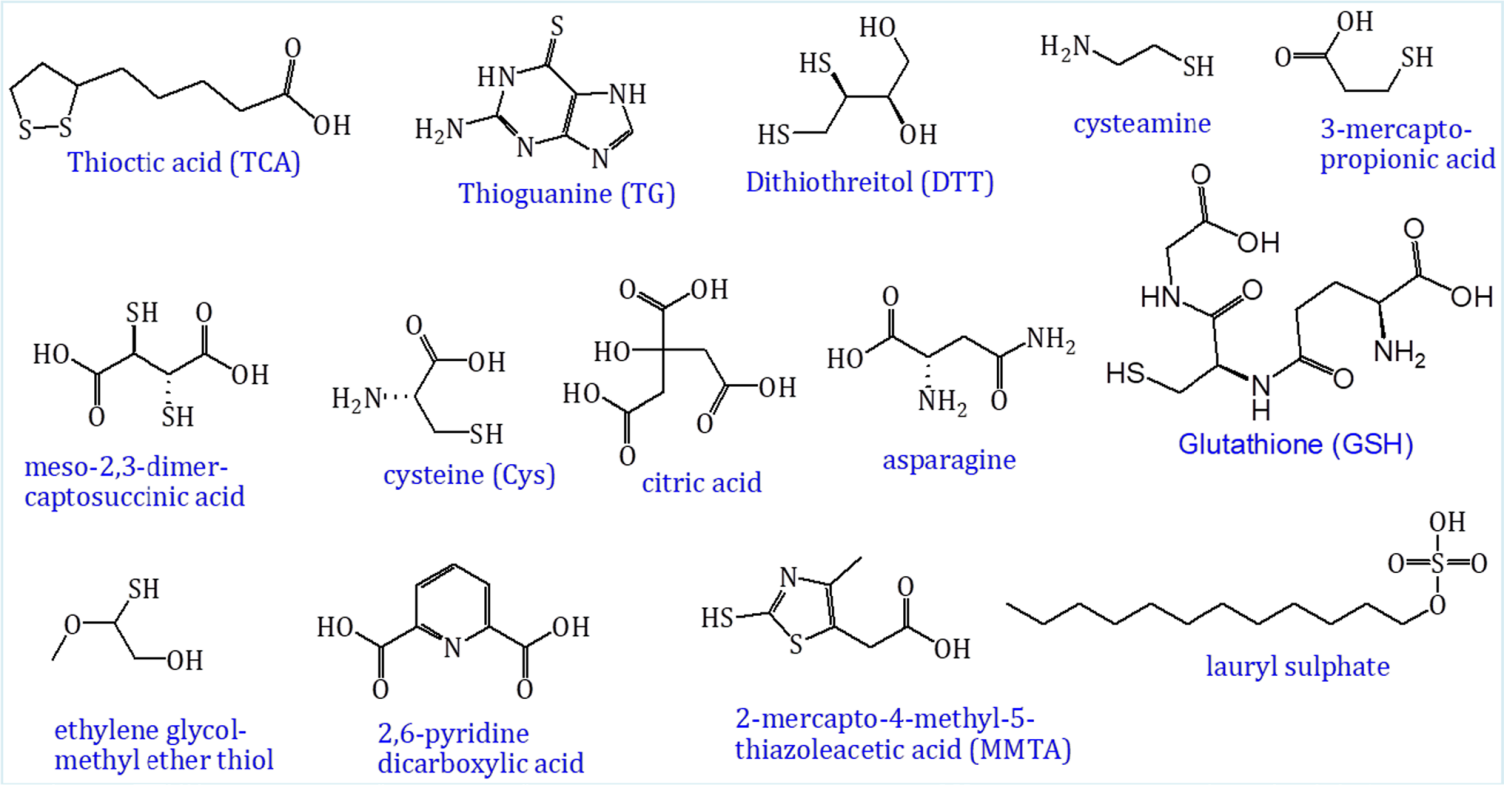
Different types of arsenic-recognition specific ligands used for the development of arsenic nanoprobes

The surface modified nanomaterials have also been widely applied for colorimetric sensing based on the specific interaction between detecting analyte and the modified ligands [137, 138]. The attachment of ligand molecules to the surface of nanomaterial can be carried out either by non-covalent interaction or covalent bound conjugation [139]. The desired properties of these nanomaterial-based colorimetric sensors for metal-ion detection, including arsenic, can be obtained by doing appropriate surface modifications in their synthesis methods because their properties are size and shape dependent [140, 141]. The chelation chemistry of arsenic with inorganic/organic ligands has been materialized for developing arsenic colorimetric sensor by simply modifying the surface of the nanomaterial with these specific ligands [142, 143]. With this interaction, the formation of the As-O, As-S or As-O-P bond results in colorimetric and catalytic properties change of these nanomaterials, which improves the sensitivity and specificity of detection performance. Different types of arsenic-binding ligands that have high supermolecular chemistry on the surface of nanomaterial are reported for the design of nanomaterial-based arsenic nanosensors. The most common surface-modified ligands used for arsenic determination are summarized in Fig. 11.

Among the various functionalized nanomaterials, gold nanoparticles (AuNPs) are the most extensively studied, and widely used for colorimetric detection [144–146]. These nanoparticles present interesting and unique optical properties due to their strong surface plasmon resonance (SPR) absorption in the visible region and their ease of operation, fabrication, and stability. Therefore, any changes in the optical properties may lead to SPR absorption and colorimetric changes. The use of surface-modified AuNPs as optical nanoprobes greatly improves the sensitivity of colorimetric sensors for arsenic monitoring due to their high extinction coefficient compared with organic dyes [123]. Various AuNPs-based arsenic colorimetric methods were reported with excellent limits of detection and good selectivity [147–149]. These optical sensors generally depend on the arsenic-induced aggregation or disaggregation of AuNPs, which results in a color change and a variation in the absorbance peak in the visible region.

**Fig. 12:**
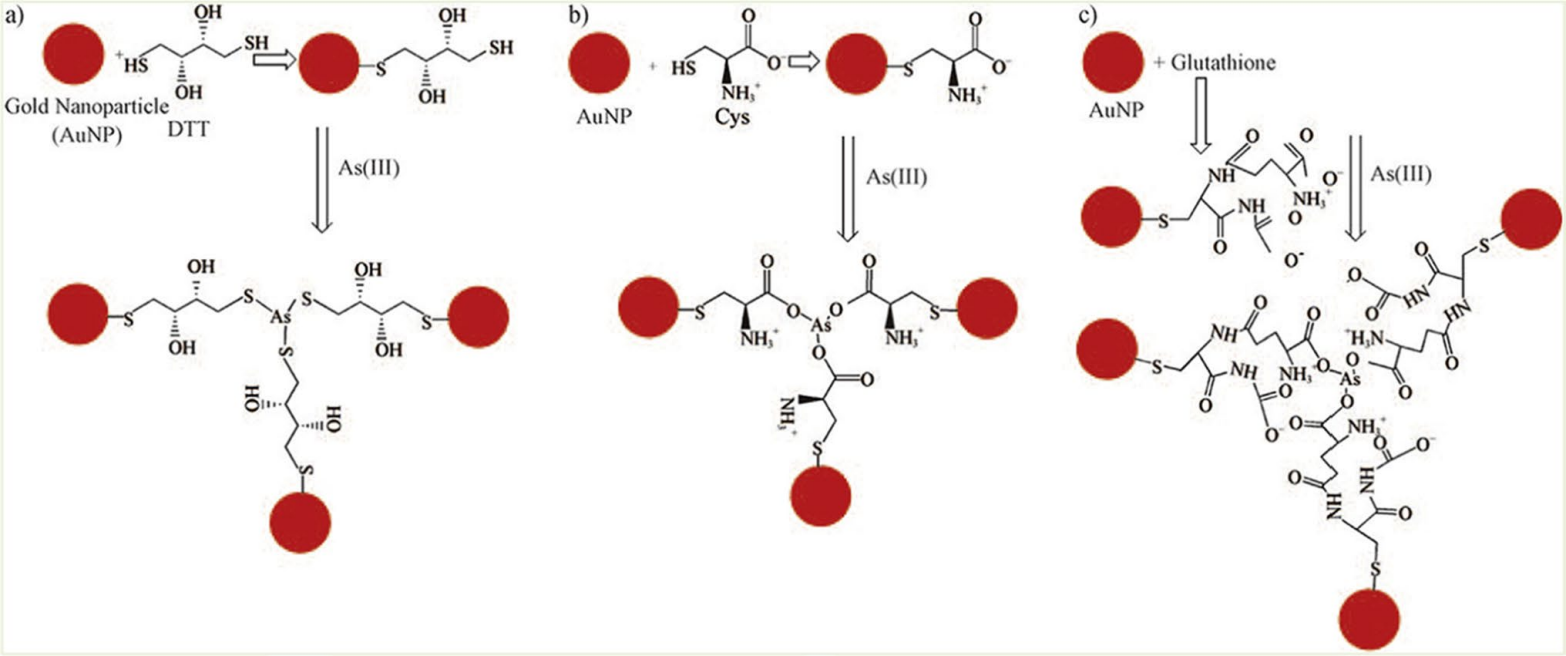
Schematic demonstration of Thiol-AuNPs based arsenic detection. **a** DTT-functionalized AuNPs, **b** Cys-functionalized AuNPs, and **c** GSH-functionalized AuNPs. This figure was obtained with permission from Ref. [147]

Thiol-gold chemistry is the most common colorimetric method used for arsenic detection [150]. Therefore, many thiol (SH)-rich ligands were frequently incorporated in AuNP-based colorimetric methods for on-site detection of arsenic due to their strong bonding between sulfur and arsenic. For instance, Kalluri et al. demonstrated a colorimetric response of glutathione (GSH), cysteine (Cys), and a dithiothreitol (DTT)-modified AuNP based dynamic light scattering (DLS) assay for the selective detection of arsenic [147]. In their study, the specific interaction of the As(III) ion with DTT, GSH, and Cys conjugated AuNPs results in nanoparticle aggregations with stability constants of 37.8, 32.0, and 29.84 logK, respectively, and good selectivity over other heavy metals. DLS analysis was further stated to improve the limit of detection down to 10 ppt. Comparatively, a minimum detection limit (5 ppb) was obtained in the case of DTT-capped AuNP due to the strong binding of the As(III) ion via the As-S bond, while in the other two cases, GSH and Cys-modified AuNPs, the As (III) ion binds through the As-O linkage due to the lack of free SH groups available for chelating (Fig. 12).

Similarly, citrate-based AuNPs functionalized with GSH-DTT-Cys have been reported for As (III) detection in the presence of 2,6-pyridinedicarboxilic acid (PDCA) as a chelating agent [148]. Additionally, a dithiothreitol-modified gold nanorod (DTT-AuNRs) has also been developed as a selective colorimetric probe for the detection of arsenic (III) in aqueous solutions [151]. The developed dithiothreitol-capped AuNRs show high sensitivity for As (III) detection with a low detection limit of 38 nM, and have been applied for As (III) sensing in environmental water samples. Interestingly, Shrivas and coworkers proposed another colorimetric sensing strategy for As (III) based on lauryl sulfate (LS)-functionalized AuNPs by utilizing the competitive interaction between AuNPs, LS, and arsenite [152]. In the presence of As_2_O_3_, As(III) would replace the LS modifying ligands from the surface of AuNPs, resulting in aggregation and the color change due to the instability of AuNPs. This colorimetric method was successfully applied for the detection of arsenite in natural water with a 2 ppb detection limit. Other than gold-thiol based reactions, different functionalized ligands, such as phosphonium ionic liguid [153], S-layer-protein [154], calix[n]arene-based supramolecule [155] and polyethylene glycol [156] have also been incorporated into AuNPs-based colorimetric sensor design for arsenic determination in real environmental samples.

**Fig. 13:**
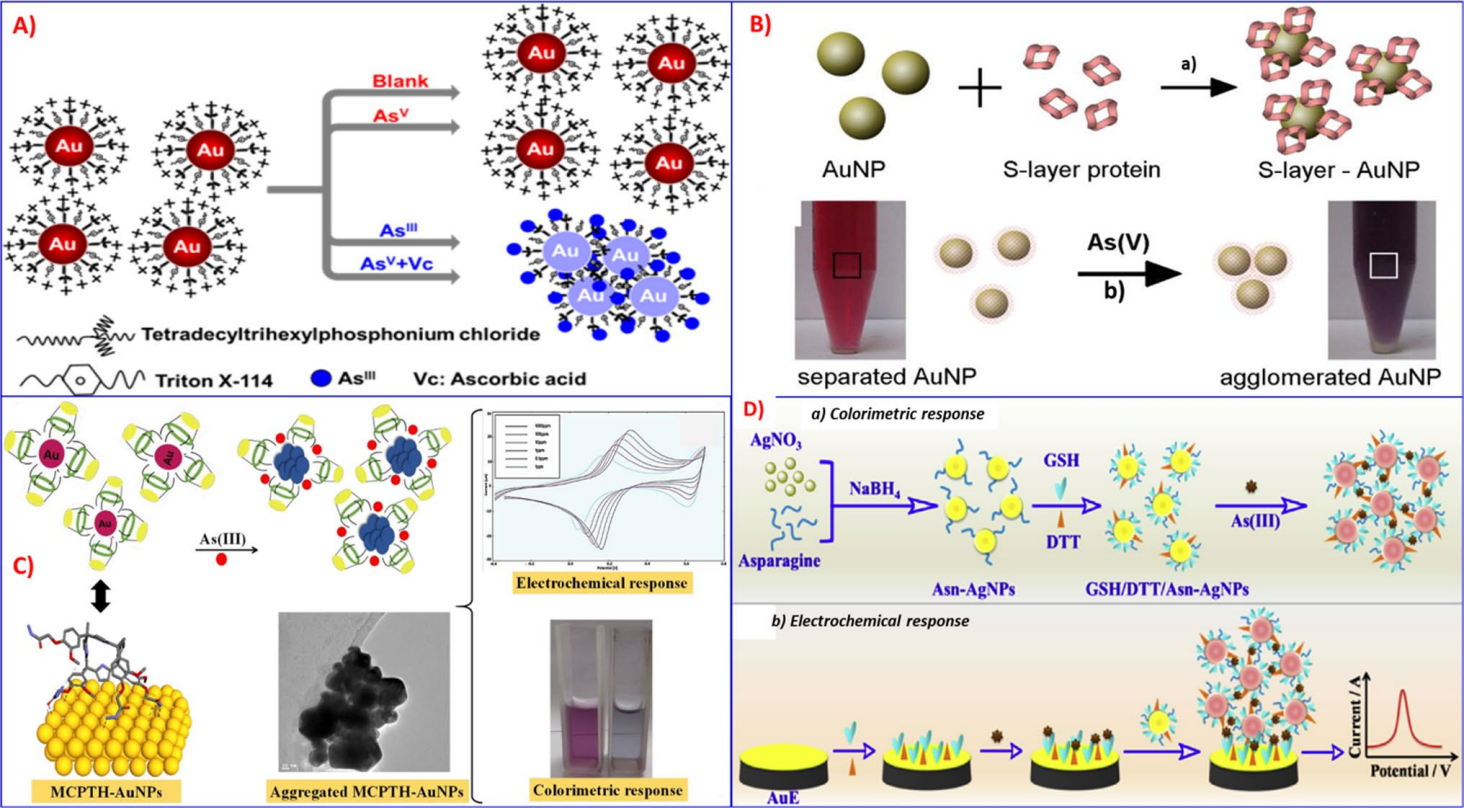
**A** Schematic representation of the mechanism of P-AuNPs for determination of arsenic, adopted from Ref. [153]. **B** schematic illustrations of S-layer-modified spherical AuNPs for arsenic detection, reproduced from Ref. [154]. **C** Schematic representation of the MCPTH-AuNPs-based dual-modal assay for arsenic detection, reproduced from Ref. [155]. **D** Schematic Illustration of the Asn/DTT/GSH-AgNPs-based dual-modal assay for arsenic detection, reproduced from Ref. [157]

Tan et al*.* developed a colorimetric C_14_(C_6_)_3_P^+^-functionalized AuNP (P-AuNP) probe for naked-eye speciation tests of As (III) and As (V) based on the specific interaction between arsenite and phosphonium ionic ligands [153]. In this method, the selective interaction between As (III) and phosphonium ionic liguid (C_14_(C_6_)_3_P^+^) was demonstrated by extended x-ray absorption fine structure (EXAFS) analysis, which shows much stronger binding to As (III) than As (V). As shown in Fig. 13A, As (III) is detected directly based on the color change of the P-AuNP probe from red to blue. As (V) concentration is determined indirectly by subtracting from the total inorganic arsenic by pre-reducing As (V) into As (III) via ascorbic acid. The developed protocol was applied for a visual speciation test of arsenite and arsenate at levels below the WHO guideline for drinking water. The proposed P-AuNP probe also featured low-cost, rapidity, no requirement of equipment, and high tolerance to common potential interference ions such as phosphate ions (PO_4_^3−^, 10 nM), which make the P-AuNP colorimetric probe suitable for the field test of As (III) in environmental samples. Arsenate concentration can be determined indirectly by subtracting arsenite concentration from the total inorganic arsenic. Lakatos et al. demonstrated the synthesis of an S-layer protein-functionalized AuNPs based colorimetric method for detecting anionic arsenate complexes in an aqueous medium [154]. This method exploits the aggregation of S-layer protein–functionalized spherical AuNPs in the presence of arsenic (V) species, and results in a color change from red-wine to blue for aggregated NPs. A lower detection limit of up to 24 ppb was achieved using this method (Fig. 13B). In another study, Boruah’s group developed a polyethylene glycol functionalized AuNPs-based colorimetric assay for As (III) with a detection limit of 5 ppb and a linear range of 5–20 ppb in the environmental sample [156].

Very recently, Kongor et al. reported a dual colorimetric and electrochemical sensor for the detection of As (III) in aqueous solution based on the non-covalent interaction of calix[4]pyrrole tetrahydrazide (MCPTH) supramolecules on the AuNP surfaces [155].

Calix[n]arene derived platforms are 3rd generation supramolcules that have been used for the design of new innovative receptors. As illustrated in Fig. 13C, the well-dispersed solution of MCPTH-capped AuNPs gets destabilized in the presence of As(III), which then results in disruption of the non-covalent interaction between MCPTH and the AuNP surface, which has almost lost its spherical shape. This is confirmed by the remarkable visual color change from pink to blue. The proposed MCPTH-capped AuNPs have also been applied for the amperometric electrochemical sensing of As(III) with a detection limit of 1 ppb.

Compared to AuNP-based colorimetric sensors, very few surface-functionalized silver nanoparticles (AgNPs) and Ag-based coordination polymers are reported for the colorimetric detection of arsenic [44, 157, 159–161]. For example, Wen et al*.* fabricated a multi-ligand-functionalized AgNPs colorimetric method for arsenic determination using asparagine (Asn) as the capping agent followed by modification with DTT and GSH [157]. A naked-eye colorimetric detection of arsenite can be released based on the arsenite-triggered aggregation of GSH/DTT/Asn-AgNPs (Fig. 13D). Moreover, the colorimetric assay of the solution phase can be converted into an electrode to realize surface-accumulated electrochemical analysis of As(III) with improved sensitivity and selectivity. Similarly, Siangproh et al*.* developed a simple colorimetric detection of arsenic based on selectively adsorbed onto ferrihydrite-coated silica gel using silver nanoplates (AgNPls) [159].

As an alternative solution to the costly Au and Ag NPs, cerium oxide (CeO_2_) and copper nanoparticles (CuNPs) have received significant attention for the fabrication of highly economical arsenic colorimetric probes [162, 163]. For example, Laghari et al. developed a highly stable ranolazine-functionalized CuNPs-based colorimetric sensor for trace level detection of arsenic (III) species in groundwater samples [163]. The synthetic method for Rano-Cu NPs is cheap, rapid, and simple to follow. It demonstrated a highly selective colorimetric sensor for As (III) detection via a visual color change from brick red to darkening green with a low detection limit of 16 nM. When these colorimetric sensing concepts and techniques are combined with appropriate nanomaterials with unique optical properties, they may provide significant performance improvements for the measurement of inorganic arsenic, such as improved selectivity, increased sensitivity, cheaper cost, faster response, and naked-eye detection. This simple design, quick response time, and easy-to-interpret function are critical to the current development of sophisticated colorimetric sensor materials, particularly for real-time and on-site applications.

## Paper-based colorimetric sensor

**Fig. 14:**
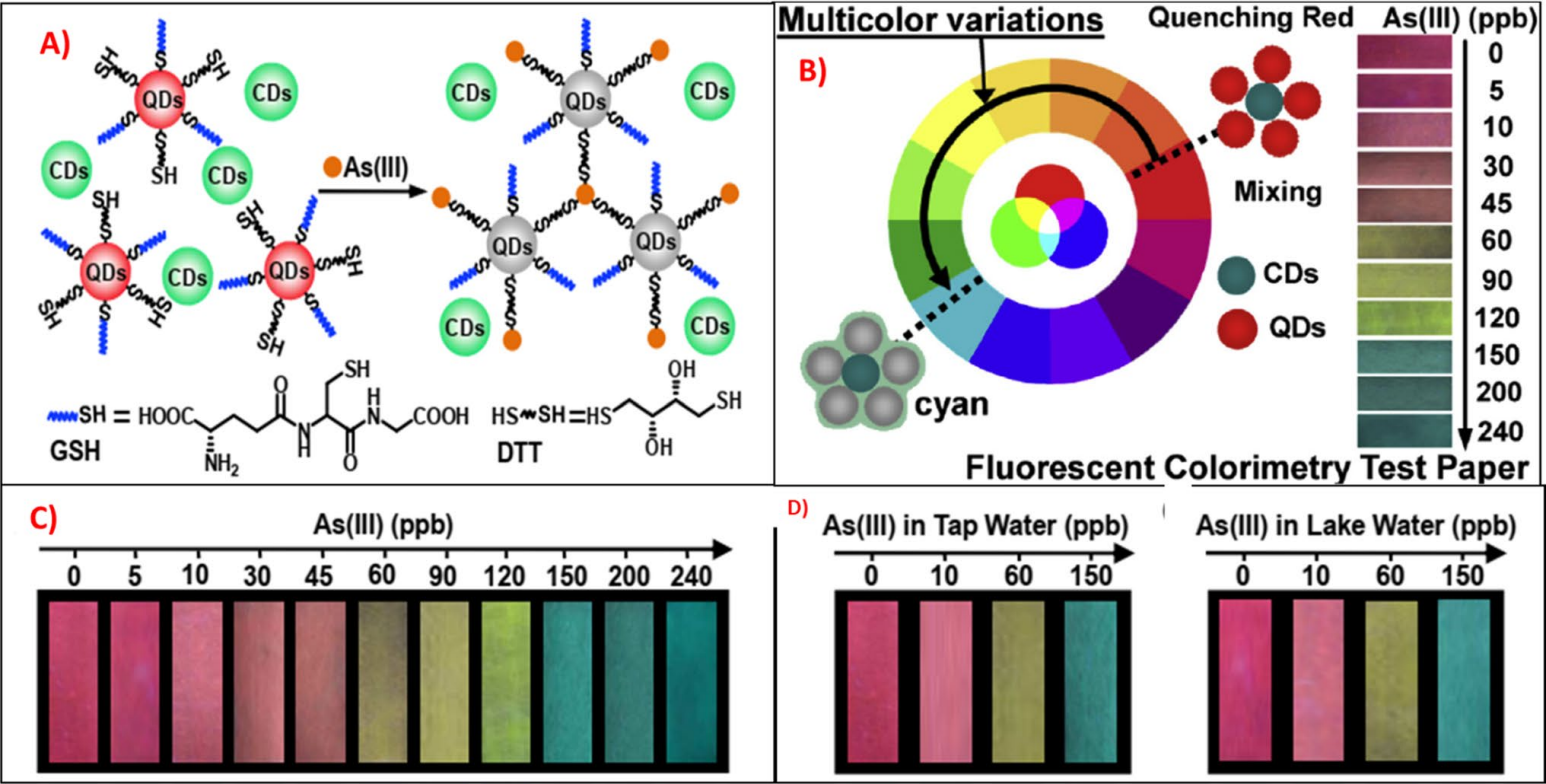
**A** The visualization mechanism of As(III) using GSH/DTT-QDs as fluorescent sensory probe and CDs as an internal standard probe; **B** proposed strategy to achieve the wide range of color variations from red to cyan by the use of dual-color probes; **C** the visualization of As(III) using the fluorescent test papers prepared by printing GSH/DTT-QDs/CDs ink on a piece of filter paper; **D** the visual detections of As(III) in tap water and lake water. This figure was obtained with permission from Ref [158]

Paper-based microfluidic devices have been extensively investigated for their potential use in optical biosensor applications [164–167]. Because of their advantages in simplicity, portability, disposability, practicability, and visual detection, colorimetric tests should be carried out on solid-state platforms for field applications [168]. Meanwhile, the sensing principles of the paper-based colorimetric assay for arsenic detection are based on the above-mentioned colorimetric sensing strategies, and they are very simple and practical but advanced colorimetric sensors [169–171]. For example, Zhang and his group reported a color-multiplexing based fluorescent colorimetry test paper with a variety of dosage-response visualizations of arsenic (III) as low as 5 ppb [158]. In their study, the red quantum dots (DQs) were functionalized with GSH and DTT to obtain excellent sensitivity to As (III) (Fig. 14A). The cyan carbon dots (CDs) with spectral blue-green components were mixed with the DQs solution to produce a red fluorescence composite. The addition of As (III) into the sensory solution resulted in the fluorescent color evolving from red to cyan gradually (Fig. 14B, C). The sensory solution was printed onto a piece of filter paper to realize dosage-sensitive visualization for the detection of arsenic in tap water and lake water (Fig. 14D).

Using similar approaches, Chanda’s group reported a paper-based nanosensor for the colorimetric detection of arsenic species using functionalized AuNPs with different capping agents such as thioguanine (TG) [172, 173], meso-2,3-dimercaptosuccinic acid (DMSA) [174], and 2-mercapto-4-methyl-5-thiazoleacetic acid (MMT) [175]. A simple paper-based microfluidic device for the detection of arsenic was reported using a gold-modified nanosensor [172].

**Fig. 15:**
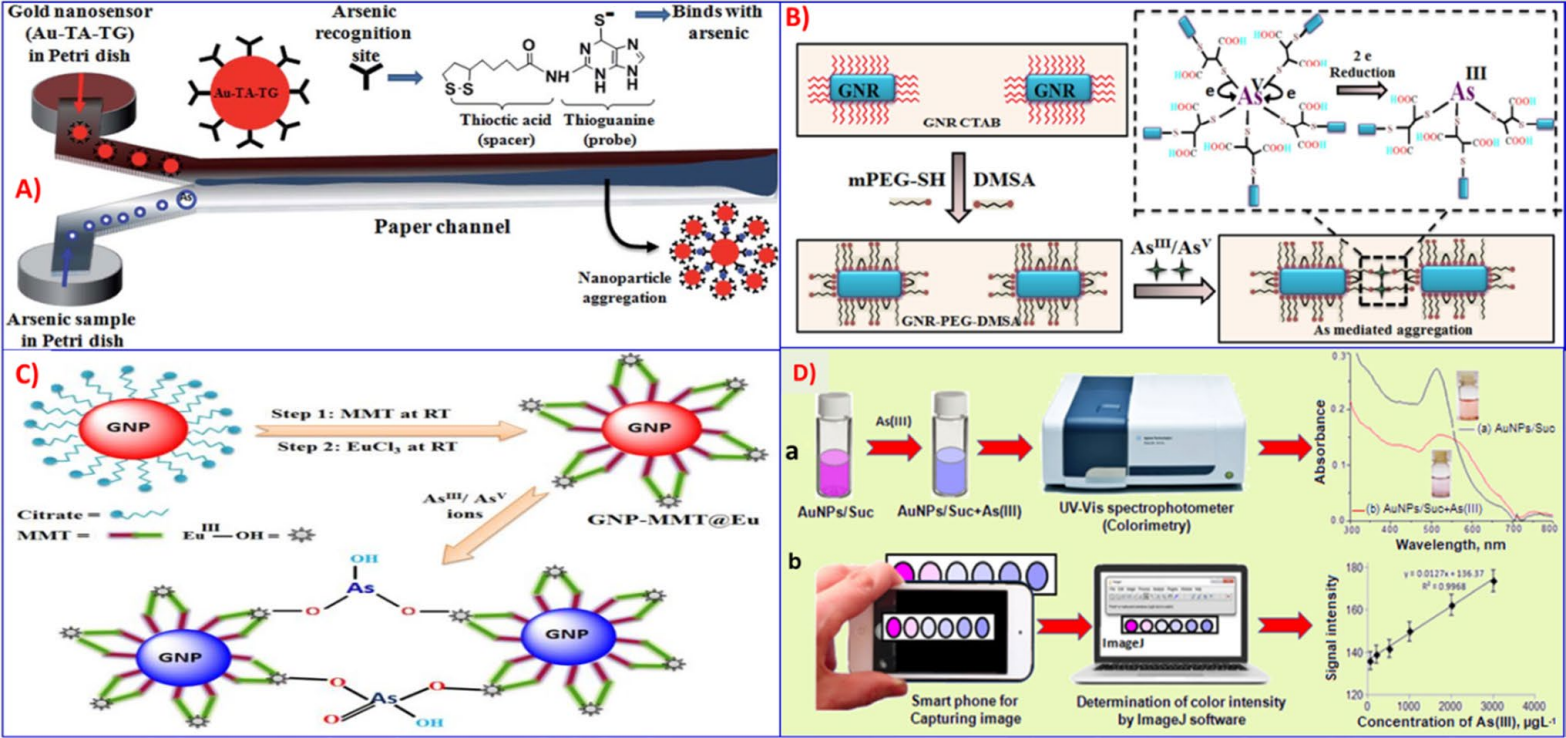
A schematic representation of paper-based microfluidic analytical device for arsenic monitoring using Au-TA-TG nanosensor **A** reproduced with permission from Ref. [172], **B** GNR-PEG-DMSA nanorods reproduced with permission from Ref. [174], **C** GNP-MMT@Eu nanosensor reproduced with permission from Ref. [175], and **D** smartphone-integrated paper device fabricated with AuNPs/Suc reproduced with permission from Ref. [176]

The proposed nanoprobe (Au-TA-TG) was prepared by step-wise chemical conjugation of AuNPs with thioctic acid (TA), followed by thioguanine (TG) ligands in the presence of conjugating agents. This method develops a visible dark bluish-back precipitate at the interface of the microchannel due to the aggregation of nanoparticles via transverse diffusive mixing of As (III) ions with Au-TA-TG (Fig. 15A). Then, the amount of arsenic originally present in the solution is related to the amount of color that precipitated. Their rapid, sensitive Y-shaped design is specific for arsenite, and can detect arsenic at very low concentration levels (1 ppb). Similarly, a gold nanorods (GNR) paper-based colorimetric sensor, GNR-PEG-DMSA, for arsenic detection was reported using GNR by conjugations with poly(ethylene glycol) methyl ether thiol (mPEG-SH), followed by DMSA [174]. As illustrated in Fig. 15B, the interaction of As (III) or As (V) ions with the free thiol group of the GNR-PEG-DMSA probe results in arsenic-induced aggregation of nanorod particles with distinct color changes from dark bluish-purple to almost colorless. The proposed assay responded to both As(III) and As(V), since arsenate can be reduced to arsenite by DMSA ligand, and thus the total arsenic in the water sample can be quantified. Moreover, this color-based response GNR-PEG-DMSA sensor is also utilized to develop a smart paper-based device that can be used for field detection of both arsenic species in real water samples with a detection limit of 1 ppb. In another study, the same group developed a GNP-MMT@Eu nanosensor for arsenic detection by modifying the AuNPs with other MMT followed by europium chloride (EuCl_3_) [175]. Both arsenite and arsenate can induce GNP-MMT@Eu aggregation through electrostatic attraction and covalent-type interaction between the As-OH/As-O^−^ groups of arsenic species and the Eu-OH/Eu-OH^2+^ of the GNP-MMT@Eu nanoprobe (Fig. 15C). This results in a visible color change from red to blue. In another approach, the GNP-MMT@Eu nanoprobe was validated for on-site detection of total arsenic using a filter paper strip and achieved almost a 0.1 ppm detection limit of total arsenic by naked eye.

In a similar case, a few research groups reported the use of a smartphone-integrated colorimetric test strip for the detection of arsenic (III) in contaminated environmental samples using sucrose-modified gold nanoparticles (AuNPs/Suc) as nanoprobes [176, 177]. For example, AuNPs/Suc is fabricated by the reduction of chloroauric acid using sucrose as a capping agent [176]. The addition of As(III) to the AuNPs/Suc nanoprobe vial results in a color change from pink to blue. The color intensity of the solution was measured using spectrophotometry in the wavelength ranges of 200–800 nm, as illustrated in Fig. 15D. To develop a smartphone-integrated paper-based device, the AuNPs/Suc nanoprobe was placed on a round paper substrate, followed by the addition of different As (III) concentrations. The color intensity of the test paper zone with As (III) was measured using IMageJ software (Fig. 15D). This smartphone-integrated paper device demonstrated a linear range of 50 to 3000 µg/L with a detection limit of 20 µg/L. Another research integrates smartphone, microfluidics, and colorimetric applications for simultaneous detection of As(III) and Hg(II) ions based on the plasmon resonance properties of gold nanoparticles. The colorimetric signals originated due to the nanoparticle aggregates formation through the interaction of gold nanoparticles with DTT-10,12-pentacosadienoic acid, and lysine in the presence of As (III) and Hg (II) ions [178]. In another study, leucomalachite green (LMG) dye was integrated into a microfluidic platform to probe the colorimetric reaction between arsenic and KI and liberate iodine, which results in LMG oxidation into malachite green under acidic media, forming a strong green color on a paper-based test strip [179]. The LOD was 0.19 ppb. Similarly, Nagy and a coworker developed a portable and semi-automated colorimetric sensor device for the determination of arsenic in drinking water with a sensitivity of 1 µg/L [166]. The greatest advantages of paper-based microfluidics analytical devices are that they are biodegradable, low-cost, and do not need any sophisticated facilities or instrumentation. They also offer rapid detection by a simple color change that could be detected by the naked-eye. Due to the above advantages, this method has attracted significant attention in analytical chemistry as a powerful tool for rapid monitoring of toxic metals, especially for remote field applications.

## Conclusion and future prospects

Contamination of drinking water by arsenic species significantly threatens human health, and the effective real-time monitoring of arsenic in the environment, however, remains a major challenge in many countries. Recently, advanced optical colorimetric sensors have emerged as a promising analytical tool for rapid monitoring of toxic pollutants, including arsenic, in both lab and field measurements due to their fast visual detection. The advantages of using simple instrumentation, portability, and ease-to-use for field application along with visual quantification have made optical sensors preferred in many areas of research as compared to the traditionally used techniques. In this review, we have discussed and reviewed the advancement of optical colorimetric sensor strategies for arsenite (As^+3^) and arsenate (As^+5^) determination in the environment. These colorimetric assays are based on chemical and molecular interactions between the surrounding materials (e.g., dye/reagent, biomolecule, polymer, and nanoparticle) and the target arsenic species, which make the overall method sensitive and specific. Furthermore, various nanomaterials with unique colorimetric and catalytic properties are discussed for sensing applications involving arsenic species. It should be noted that the development of colorimetric methods combines chemical, biological, and nanomaterials to endow them with better sensing and analytical performance. In addition, the use of paper material for point-of-care analysis greatly advanced the colorimetric sensing of arsenic for on-site field applications. The types of sensing, materials/probes, and detection limit of various high sensitive optical colorimetric sensors of inorganic species are summarized in Table 1.

**Table 1:** Summary on the type of sensing, materials/probes, and detection limit of various high sensitive optical colorimetric sensors of inorganic species

Type of sensing	Materials/probes	Analyte	Limit of detection	References
Redox-dye chemistry	Norbornene polymer	As(III)	1.2 ppb	[87]
	Rhodamine-B (RhB)	As(III)	0.64 ppb	[88]
	Benzothiazole Schiff-based probe	As(III) As(V)	7.0 ppb	[89]
	MB-based Fenton-like reaction	As(V)	0.358 ppb	[90]
Nanozyme-based sensor	CoOOH nanoflakes	As (V)	3.72 ppb	[110]
	FeOOH nanorod	As(V)	0.1 ppb	[111]
	Fe-Co-LDH@3-MPA	As(III)	35 ppb	[112]
	3D flower-like iron alkoxide	As(V)	1.57 µg/L	[117]
	Pd-DTT	As(III)	35 ng/L	[114]
	Octahedral Mn_3_O_4_ NPs	As(V)	1.32 µg/L	[115]
Sensor-based on aptamer	PDDA-induced aggregation of AuNPs	As(III)	5.6 ppb	[125]
	CTAB-induced aggregation of AuNPs	As(III)	0.6 ppb	[126]
	short ssDNA@AuNPs	As(III)	0.18 ppb	[131]
	CNT_COOH_-BSA hybrid system	As(III)	6 μg/L	[132]
Ligand-tethered nanosensors	phosphonium ionic liguid (C_14_(C_6_)_3_P^+^)	As(III), As(V)	7.5 μg/L	[153]
	S-layer-modified spherical AuNPs	As(III)	24 ppb	[154]
	calix[4]pyrrole-tetrahydrazide supramolecules	As(III)	1 ppb	[155]
	GSH/DTT/Asn-AgNPs	As(III)	0.36 ppb	[157]
Paper-based colorimetric sensor	Au-TA-TG nanosensor	As(III)	(1 ppb	[172]
	GNR-PEG-DMSA	As (V)	1.0 ppb	[174]
	GNP-MMT@Eu	As (V)	1.0 ppb	[180]
	AuNPs/Suc	As(III)	20 µg/L	[176]

Despite the above achievements, key challenges also exist in the exploration of these arsenic colorimetric sensing principles and strategies for practical or commercial application in the following aspects: (i) currently, most of the reported arsenic colorimetric optical sensors are only verified in the lab or in simple sample matrices. Thus, more validation experiments should be carried out for monitoring toxic arsenic in various real environmental samples such as soil, plants, wastewater, etc. (ii) Speciation of inorganic arsenite and arsenate. The toxicity of arsenic largely depends on its oxidation state. Typically, arsenite is more toxic than arsenate. So far, many of the reported colorimetric probes cannot directly differentiate the two arsenic species (As^+3^ and As^+5^) effectively. Hence, speciation analysis of inorganic arsenic is important for species migration and transformation analysis, as well as toxicity evaluation. Moreover, these strategies are also limited to detect organic arsenic compounds such as MMA and DMA. Thus, additional conversion and separation procedures are still important to be coupled with these advanced methods. It is believed that developing advanced nanosensing strategies with unique colorimetric properties to directly analyze different forms of arsenic species will be an important research area in the future. (iii) On-site Analysis. The development of advanced emerging analytical tools for the analysis of toxic arsenic in field applications is also another major challenge. To obtain accurate pollution-cause information, rapid monitoring of arsenic at the pollution-site is particularly important. Among the reported methods, fortunately, paper-based colorimetric sensors can make rapid detection of arsenic at pollution-site come true. In addition, it will be an important research area in the future. (iv) Interference from the environmental sample. The reported optical colorimetric sensors are not selective or specific enough to detect arsenic in complex sample matrices. The presence of iron, phosphate, and sulfur-containing compounds in the sample matrices has been found to cause potential interference in these colorimetric assays. This limits the wide use of arsenic colorimetric sensors in the analysis of real environmental samples and may require additional separation or concentration of arsenic compounds from the sample matrices before the detection step.

To overcome these obstacles, more efforts need to be dedicated to develop cost-effective colorimetric-based arsenic nanosensors for practical use or commercialization. Authors conclude that future work will continue to explore different advanced nanomaterials for on-site arsenic determination with simpler instrumentation, faster response, practicability, higher sensitivity, easy visual color detection, and better selectivity.

## Data Availability

Not applicable.

## Abbreviations

AAS: Atomic absorption spectroscopy
ABTS: 2,2′-Azino-bis(3-ethylbenzthiazoline-6-sulfonicacid)
AFS: Atomic fluorescence spectrometry
ATP: Adenosine triphospahate
AgNPs: Silver nanoparticles
Au NPs: Gold nanoparticles
CNTs: Carbon nanotubes
CuNPs: Copper nanoparticles
DMA: Dimethylarsenic acid
EEA: Electrophorese electrochemical assay
EPA: Environmental Protection Agency
ICP-MS: Inductive couple plasma mass spectrometry
LOD: Limit of detection
MMA: Monomethylarsenic acid
TMB: 3,3′,5,5′-Tetramethylbenzidine
WHO: World Health Organization
